# Fixed combination of palmitoylethanolamide and melatonin in preventive therapy of migraine: results from a randomized clinical trial

**DOI:** 10.3389/fnut.2025.1560654

**Published:** 2025-04-10

**Authors:** Vincenzo Piccolo, Adua Marzocchi, Maria Maisto, Vincenzo Summa, Gian Carlo Tenore, Angela Amoresano

**Affiliations:** ^1^Department of Pharmacy, University of Naples Federico II, Naples, Italy; ^2^Interuniversity Consortium of Biostructures and Biosystems (INBB), Rome, Italy; ^3^Department of Chemical Sciences, University of Naples Federico II, Naples, Italy

**Keywords:** migraine, palmitoylethanolamide, melatonin, supplement, clinical trial

## Abstract

**Introduction:**

Migraines are neurological disorders which significantly impact quality of life. Current pharmacological treatments often have adverse effects, prompting the search for alternatives with fewer side effects. Several studies have described the antimigraine properties of palmitoylethanolamide (PEA) and melatonin.

**Materials and methods:**

Our research assessed the efficacy of the association of hydrodispersible PEA (1,200 mg) and melatonin (0.2 mg) by a randomized, three-month, double-blind, placebo-controlled trial (PEATONIDE^®^; *n* = 30 patients; placebo; *n* = 30 patients). The participants were recruited by “I.N.B.B. Consortium” in Italy. The primary outcome was the reduction of migraine frequency, while secondary outcomes included the reduction of intensity, duration, and grade of disability. The parameters were assessed by a self-reported daily headache diary.

**Results:**

The formulation displayed a significant reduction in frequency (T3 months: 2.2 ± 0.4 MMDs; T0 baseline: 3.4 ± 0.5 MMDs, ***p < 0.001 *vs* baseline T0) and duration, intensity, disability, and incidence of associated symptoms of migraine attacks after 3 months of treatment. No adverse effects were observed during the treatment. In addition, a significant mitigation of migraine-related symptomatology was observed.

**Conclusion:**

These findings suggest that PEATONIDE^®^ may be a promising adjunctive approach for migraine management. However, given the relatively small sample size, further large-scale and multicenter trials are needed to confirm its clinical applicability in broader migraine population.

## 1 Introduction

Migraine represents a chronic and severe neurological condition, causing significant debilitation and pain for about 15% of the global population (1). According to the International Classification of Headache Disorders, chronic migraine is defined as experiencing headaches on 15 or more days per month for over 3 months, with at least 8 days per month meeting the criteria for migraine (Monthly Migraine Days, MMDs), including pulsating quality, moderate to severe intensity, nausea, or sensitivity to light and sound. In contrast, episodic migraine is characterized by headaches occurring on fewer than 15 MMDs. Migraine is further classified into high-frequency episodic migraine (HFEM), with 8 to 14 MMDs, and low-frequency episodic migraine (LFEM), with fewer than 8 MMDs (2, 3). Migraine significantly affects the quality of life, ranking among the top 20 causes of disability in the adult population according to the World Health Organization. Despite the considerable impact on quality of life and decline in work performance and productivity, migraine often goes undiagnosed and untreated (4). Migraine episodes entail intense, throbbing pain in the head and facial regions, often accompanied by sensitivity to sound (phonophobia) and light (photophobia). Around 20–25% of migraine sufferers experience visual disturbances known as aura preceding the onset of a migraine attack during the prodrome phase. Following this phase, migraine attack ensues symptoms like pain, throbbing, nausea, and heightened sensitivity to environmental stimuli (5). This phase can last anywhere from 4 to 72 h before the pain subsides, followed by a postdrome phase characterized by fatigue and cognitive impairment.

Different oral drugs are employed for the preventive treatment of migraine, including antiepileptic drugs, antidepressants, beta-blockers, calcium channel antagonists, and serotonin antagonists. These pharmacological treatments for migraine prevention are non-specific drugs, exhibiting a considerable rate of therapeutic failure, which can reach as high as 67% (6). Additionally, these medications are linked to numerous side effects (7). More specific drug treatments have been approved in recent years, including inhibitors of the calcitonin gene-related peptide (CGRP) pathway. They include several monoclonal antibodies blocking CGRP (e.g., Eptinezumab, Fremanezumab, and Galcanezumab) or its receptor (e.g., Erenumab) and small inhibitors of the CGRP receptor (e.g., Rimegepant and Atogepant). Although these drugs have greater selectivity and fewer side effects, their use is limited to patients with several monthly migraine attacks with significant disability (8). Although pharmaceuticals and biologics are commonly utilized to manage migraine symptoms, there’s an increasing interest in exploring safe and efficient complementary and alternative therapies for migraine management, especially for LFEM patients who cannot use innovative CGRP inhibitors for the treatment of this condition. These interventions encompass lifestyle adjustments, behavioral modifications, and dietary changes, either as supplements to traditional treatments or as alternatives to reduce medication dependency. Research suggests that over 40% of the U.S. population utilizes alternative migraine therapies, including dietary supplements (1). Moreover, natural compounds have always been studied as bioactive molecules for the prevention and treatment of several diseases for which, up to date, there are no resolving drug treatments (9–12).

Palmitoylethanolamide (PEA) is an endogenous lipid amide formed by ethanolamide and palmitic acid, known for its role in regulating pain and inflammation. Numerous studies have demonstrated its effectiveness in various pain disorders, along with its excellent tolerability profile (13–16). PEA anti-inflammatory activity is strictly related to an increased activation of the receptor peroxisome proliferator-activated receptor (PPAR) alpha, determining a strong neuroprotective activity (17). PEA reduces NF-kB activation, thereby lowering the transcription of pro-inflammatory cytokines associated with neurogenic inflammation, a key factor in migraine physiopathology (18). Additionally, PEA inhibits mast cell degranulation, helping to prevent inflammatory mediator release, which is relevant to migraine due to mast cell involvement in its pathogenesis (19, 20). Hence, several anti-inflammatory mechanisms have been recognized that could offer specific benefits in migraine treatment. In addition, several clinical trials demonstrated PEA efficacy in migraine treatment with a reduction of migraine events and disability without treatment-related side effects (7, 21–23).

Melatonin is a hormone with a methoxyindole structure, synthetized and secreted by the pineal gland at night under normal light/dark conditions (24). The primary physiological role of melatonin, whose secretion adjusts to night length, is to convey evidence concerning the daily cycle of light and darkness to body systems. This signaling regulates functions influenced by changes in photoperiod, such as the seasonal rhythms (25). Headache disorders, particularly migraine, are strictly related to sleep disturbances and circadian dysregulation, even if their relationship is complex and multifactorial (26). The pathophysiological mechanisms linking sleep and migraine involve the glymphatic system, which plays a role in the clearance of neurotoxic waste products from the brain during sleep, and the monoaminergic nuclei of the brainstem, which are involved in the regulation of pain and sleep–wake cycles (27, 28). Additionally, disturbances in circadian rhythms may interact with neurotransmitter pathways (e.g., serotonin, melatonin) that are implicated in migraine pathogenesis. However, the indirect chronobiological association is a clear indication of a relationship between melatonin and migraine. Several studies provided evidence about the beneficial effect of melatonin administration on headache disorders (25). Moreover, melatonin regulates several pathways involved in migraine pathogenesis, including GABA, opioid, serotonergic, adrenergic, cholinergic, and melatonergic receptors (29, 30). In addition, neurogenic vasodilation and brain inflammation represent trigger factors for migraine genesis (25). Melatonin decreases the release of vasoactive compounds (e.g., nitric oxide (NO), and CGRP) and controls mast cell degranulation, ameliorating neurogenic vascular inflammation (31, 32). In addition, melatonin determines multiple neuroprotective activities, including the control of the signal transduction mechanism of pituitary adenylate cyclase-activating peptide (PACAP), a neurotransmitter involved in migraine attacks (33, 34), the reducing of excitotoxicity and radical formation, and the downregulation of proinflammatory cytokines (e.g., TNF-*α*, IL-1β) (35).

Migraine pathophysiology is associated with mast cells, which act as the immediate call center of the neuroimmune system. They represent both the source and target of several neuropeptides which are involved in migraine inflammatory process (e.g., histamine, serotonin, NO, prostaglandins, and leukotrienes) (20, 34). Therefore, mast cell degranulation inhibitors represent an innovative therapeutic approach for the prevention of migraine attacks. It is well reported that PEA could contrast the immune response by down-regulating mast-cell degranulation (36). In addition, melatonin has a regulatory activity by inhibiting mast cells degranulation, probably by an autocrine mechanism (37). Given the common mechanisms of action of PEA and melatonin as anti-inflammatory and mast cell degranulation inhibitors, it is possible to hypothesize that a combination of the two compounds could inhibit the pathophysiological patterns underlying migraine, reducing its severity and related symptoms. Therefore, this study aimed to assess the efficacy and tolerability of a patented combination treatment (PEATONIDE^®^), containing 1,200 mg of hydrodispersible PEA and 0.2 mg of melatonin, administered once daily to patients with episodic migraine. This dosage was selected based on literature data about the antimigraine properties of PEA as single component per 1,200 mg administration (38) and the inhibition of mast-cell degranulation by PEA and melatonin combination at the dosage of 1,200 mg of PEA and 0.2 mg of melatonin (20). The study evaluated the treatment’s effects on the duration and intensity of migraine attacks, as well as the frequency of events and associated disability symptoms.

## 2 Materials and methods

### 2.1 Study design and inclusion criteria

This study was designed as a randomized, three-month, double-blind, placebo-controlled trial. A total of 70 participants were recruited from “I.N.B.B. Consortium” (Istituto Nazionale Biostrutture e Biosistemi Consorzio Interuniversitario) in “COMEGEN” (Cooperativa di MEdicina GENerale) Medical Cooperative operating within the ASL Naples 1 in the period of 16/10/2023 and 16/11/2023. The study was conducted in accordance with the Declaration of Helsinki and approved by the Ethical Committee of Azienda Sanitaria Locale Napoli 1 Centro, Naples, Italy (Project identification code 1644, approval date 20/09/2023), and all adults gave written informed consent. This study is listed on the ISRCTN registry,^1^ registered with the acronym AMSPM and accessed with ID ISRCTN52370199.^2^ The data are stored in a database at the Department of Pharmacy, University of Naples “Federico II,” 80,131 Naples, Italy. Criteria for inclusion included both men and women with an age of 18–65 years with a diagnosis of episodic migraine (fewer than 14 MMDs) with or without aura, for at least 1 year prior to recruitment, and experiencing at least two migraine attacks for at least 3 months before recruitment. The diagnosis was performed using a validated questionnaire in Italian language according to the International Classification of Headache Disorders, 3rd Edition (ICHD-3) for migraines reported by the International Headache Society (39). Exclusion criteria included chronic past and/or current alcohol use (> 14 alcoholic drinks per week), allergy or hypersensitivity to any ingredients of the active or placebo formulation, pregnancy, lactation, attempts to conceive, inconsistent supplement intake, regular use of analgesic drugs for more than 12 MMDs, and treatment with other supplements or drugs potentially that might have preventive effects on migraine. Patients were instructed to maintain their usual dietary and lifestyle habits throughout the study to minimize potential confounding effects. A 2 months run-in period was conducted to collect information on physical activity, caffeine consumption, and dietary habits that could influence migraine frequency. No changes in methodology, including eligibility criteria, were made after trial commenced. Informed consent was obtained from all subjects involved in the study. During this study, the emergence of any exclusion criteria led to an immediate termination of the participant’s involvement in the trial. A total of 10 patients were found to be ineligible for the study due to not meeting inclusion or exclusion criteria. The sample size was evaluated using G*power (v3.1.9.7) for a two-tailed independent t-test comparing MMDs reduction between groups. A power of 0.95 and a two-sided *α* error probability of 0.05 were set to minimize the probability of Type I and Type II errors. An effect size (Cohen’s d) of 0.8 was assumed for the calculation (40). The minimum required sample size was calculated as 28 participants per group (*n* = 56). Taking into account potential dropouts, we increased the final sample size to 30 participants per group (*n* = 60) to ensure sufficient power for statistical comparisons of primary and secondary outcomes.

### 2.2 Randomization and intervention

**Figure 1:**
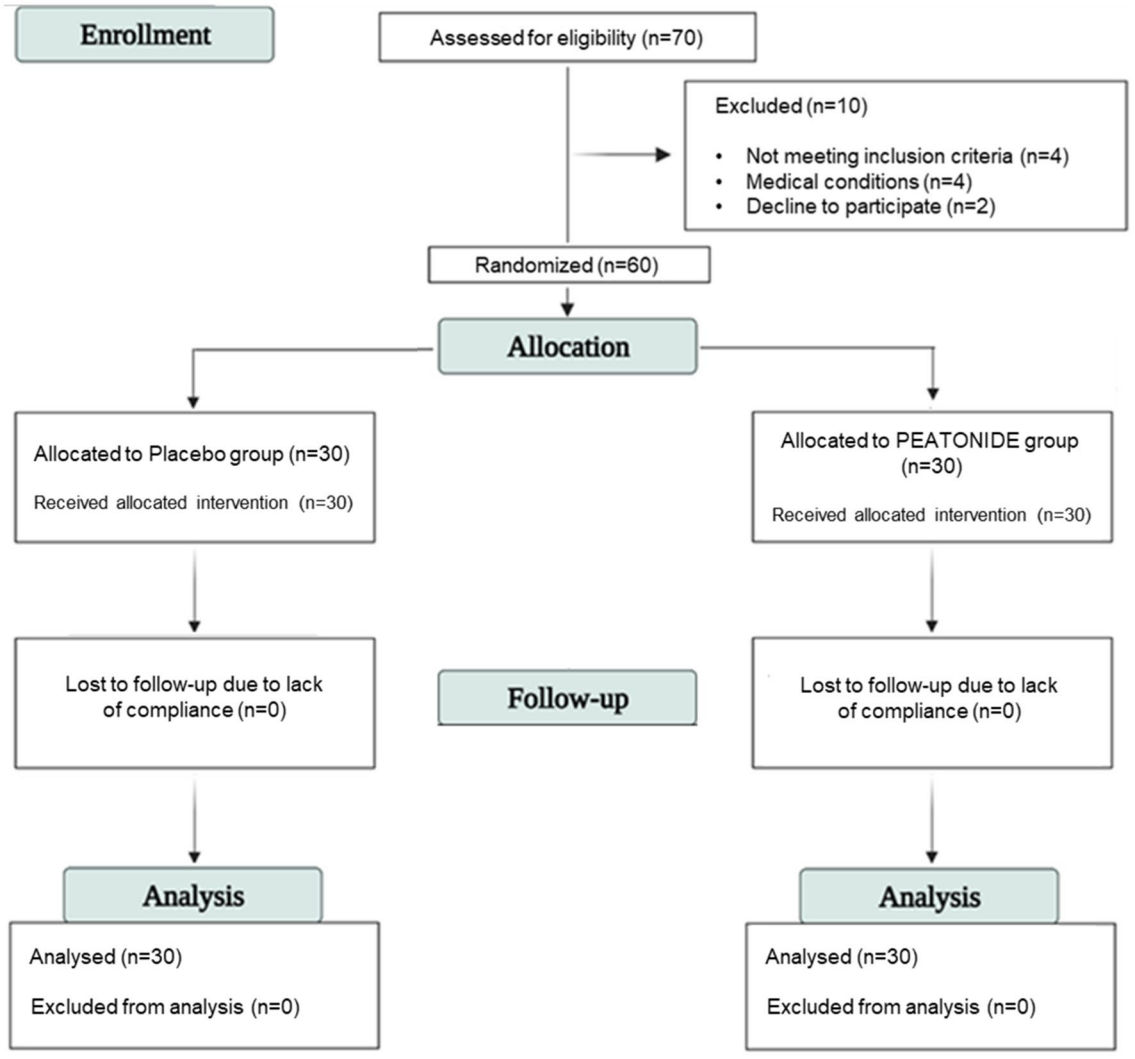
Study consolidated standards of reporting trials (CONSORT) flow diagram.

Sixty patients received the allocated intervention and completed the study. All included patients were randomly allocated into two groups (allocation ratio 1:1) and treated with a formulation consisting of 1,200 mg of hydrodispersible PEA and 0.2 mg of melatonin (PEATONIDE^®^; *n* = 30 patients) or with placebo (*n* = 30 patients) by oral self-administration taken every evening at bedtime for 3 months. Placebo and supplements were coded with different colors and given in random order. The code was not broken until all analyses were completed and the statistical analysis of the results was performed. The random list and the allocation concealment was performed by an investigator not involved in the clinical trial by using a computer generator for casual numbers. Clinicians, patients, and laboratories and trial staff (statisticians, data analysts) were blind to treatment allocation. Patients included were allowed to use analgesics limited to acetaminophen and NSAIDs as needed during the acute phase of migraine attacks. No other preventive or acute treatments were allowed during the participation in the study. The evaluation of migraines was based on the filling out of a self-reported daily headache diary by the patients. The headache diaries were administered for 2 months run-in period (T-1 and T0) and 3 months of treatment (T1, T2 and T3). After the two-month run-in period, the patients were randomly assigned to placebo or treated (PEATONIDE^®^) groups for the next 3 months. Data from the second month of the run-in period were averaged and served as baseline values. No missing data were recorded in the daily headache diaries, as all patients completed the required documentation throughout the study. A schematic summary of the stages of enrollment and patients randomization is provided by the Study Consolidated Standards of Reporting Trials (CONSORT) flow diagram (Figure 1). In the run-in and treatment periods, the following variables were collected: MMDs in the previous month; MMDs taking any analgesics in the previous month; intensity, duration and grade of disability of migraine attacks; presence of aura; presence of associated symptoms (nausea, vomiting, photophobia, and phonophobia). Intensity and disability of migraine attacks were rated on a 10-point and a 3-point Likert scale, respectively. The categorical variables were summarized in frequencies and percentages, while the numerical ones were calculated in averages and standard deviations (SD).

### 2.3 Outcome measures

#### 2.3.1 Primary and secondary outcomes

The primary efficacy outcome was the assessment of migraine frequency measured as MMDs comparing the two-months run-in with the three-month treatment period. Secondary endpoints included the reduction in intensity, duration, grade of disability of migraine attacks, the amount of analgesics used during migraine attacks, and incidence of associated symptoms of migraine attacks (e.g., nausea, vomiting, photophobia, and phonophobia). No changes in trial outcomes were performed after trial commencement. These parameters were evaluated through the completion of a self-reported daily headache diary by the patients. Duration (hours per day) and frequency (MMDs) of migraine attacks were summarized in averages, while intensity of migraine attacks and disability were rated on a 10-point (1–2 = mild; 3–4 = medium; 5–6 = strong; 7–8 = very strong; 9–10 = unbearable) and a 3-point (1 = mild; 2 = moderate; 3 = severe) Likert scales, respectively.

#### 2.3.2 Tolerability and safety

Patients were monitored for vital signs and adverse events to assess tolerability and safety. An adverse event was defined as any medical occurrence reported by a patient or noted by a clinician during the study, regardless of its suspected causes. Adverse events were recorded if they were related to the study medication. Tolerability measures included the incidences of adverse events, including gastrointestinal tract tolerance and those that led to premature withdrawal of the study and serious adverse events, including death, disability, life-threatening, and hospitalization.

### 2.4 Statistics

The categorical variables were summarized in frequencies and percentages, while the numerical ones were reported as mean ± standard deviation (SD). Statistical analyses and graphics were performed using SPSS version 26 statistical package (SPSS Inc., Chicago, USA) or GraphPad Prism (8.3.0). The results were first tested for normality to evaluate the data distribution for the assessing of the appropriate statistical test. Differences between groups were evaluated using the two-tailed unpaired Student’s t-test with Bonferroni corrections for parametric data (duration, intensity). Non-parametric data (MMDs, disability, incidence of associated-migraine symptoms) that could not meet the criteria of variance homogeneity (Levene’s test) and normal distribution (Kolgoromov-Smirnov test) were analyzed by the Wilcoxon-Man-Whitney test with Bonferroni corrections. The threshold of significance (two-tailed *α*-value) was set at 95% (*p* < 0.05).

## 3 Results

### 3.1 Clinical benefits of PEATONIDE^®^ in migraine management

Sixty patients (30 women and 30 men) were recruited (16/10/2023–16/11/2023), with a mean age of 42.5 ± 10.5 years (95% CI 38–46). The population is equally distributed between females and males. All patients included had a diagnosis of episodic migraine (with or without aura) for at least 1 year before recruitment and experienced at least two migraine attacks per month for at least 3 months before recruitment. Of all patients included, 27% had a diagnosis of episodic migraine with aura (*n* = 8 in placebo group and *n* = 8 in PEATONIDE^®^ group) and 73% had a diagnosis of episodic migraine without aura (*n* = 22 in placebo group and *n* = 22 in PEATONIDE^®^ group). In addition, the study population includes patients with low-frequency episodic migraine, with a baseline value of 3.4 ± 0.5 MMDs. After the 2 months run-in period for assessment of baseline parameters, patients were divided into two groups, PEATONIDE^®^ treated (*n* = 30 patients) and placebo (*n* = 30 patients). The mean age in PEATONIDE^®^ group was 43 ± 11 years (95% CI 39–47), while in placebo group the mean age was 42 ± 10 years (95% CI 38–46) The enrolled patients took PEATONIDE^®^ or a placebo every evening at bedtime for 3 months. None of them left the study before the end of the clinical trial and adverse effects were not reported in any of the study participants. Data concerning general and observational characteristics of the patients at the baseline period and every month of treatment are summarized in Table 1. At the end of data analysis, the clinical trial was finished (16/04/2024). These data were collected by filling out a daily headache diary by the patients. The nominal data were obtained using a rating scale.

**Table 1:** General data and biochemical parameters of placebo and PEATONIDE^®^ groups at run-in (T-1), baseline (T0) and during 3 months of treatment (T1, T2, and T3).

Parameters	Placebo (*n* = 30)					PEATONIDE^®^ (*n* = 30)				
	T-1	T0	T1	T2	T3	T-1	T0	T1	T2	T3
Male, *n* (%)			16 (54%)					14 (46%)		
Female, *n*° (%)			14 (46%)					16 (54%)		
Age (years)			43 ± 11					42 ± 10		
Type of migraine										
Without aura	22 (73%)	22 (73%)	22 (73%)	22 (73%)	22 (73%)	22 (73%)	22 (73%)	22 (73%)	29 (97%) **	30 (100%) ***
With aura	8 (27%)	8 (27%)	8 (27%)	8 (27%)	8 (27%)	8 (27%)	8 (27%)	8 (27%)	1 (3%) **	0 (0%) ***
Days of headache per month	2.9 ± 0.3	2.8 ± 0.4	3.0 ± 0.8	2.9 ± 0.7	3.0 ± 0.4	3.6 ± 0.6	3.4 ± 0.5	3.4 ± 0.5	2.8 ± 0.5 *	2.2 ± 0.4 ***
Days of analgesic use	2.9 ± 0.3	2.8 ± 0.4	3.0 ± 0.8	2.9 ± 0.7	2.8 ± 0.6	3.6 ± 0.6	3.4 ± 0.5	3.4 ± 0.5	2.8 ± 0.5 *	2.2 ± 0.4 ***
Intensity of migraine attack	6.5 ± 1.1	6.4 ± 1.3	6.5 ± 1.2	6.5 ± 1.2	6.5 ± 1.1	7.7 ± 0.9	7.6 ± 0.9	7.4 ± 0.8	6.0 ± 0.7 *	4.9 ± 0.7 ***
Duration of migraine attack (hours)	9.9 ± 0.9	9.9 ± 0.8	9.9 ± 0.9	9.9 ± 0.7	9.9 ± 0.7	10.0 ± 1.1	10.0 ± 1.1	8.9 ± 1.3*	8.4 ± 1.6 *	7.1 ± 1.7 **
Nausea incidence (%)	26 (87%)	26 (87%)	26 (87%)	26 (87%)	26 (87%)	28 (93%)	28 (93%)	28 (93%)	24 (80%) *	10 (33%) ***
Vomiting incidence (%)	15 (50%)	15 (50%)	14 (47%)	15 (50%)	15 (50%)	15 (50%)	14 (47%)	14 (47%)	4 (13%) **	0 (0%) ***
Photophobia/phonophobia incidence (%)	27 (90%)	26 (87%)	26 (87%)	26 (87%)	26 (87%)	27 (90%)	28 (93%)	28 (93%)	26 (87%)	22 (73%) *
Moderate–severe disability (%)	23 (77%)	23 (77%)	21 (70%)	23 (77%)	23 (77%)	28 (93%)	28 (93%)	28 (93%)	24 (80%) *	9 (30%) ***

Different symbols reveal significant differences compared to run-in baseline period T0 (*p < 0.05 vs T0; **p < 0.01 vs T0; ***p < 0.001 vs T0).

**Figure 2:**
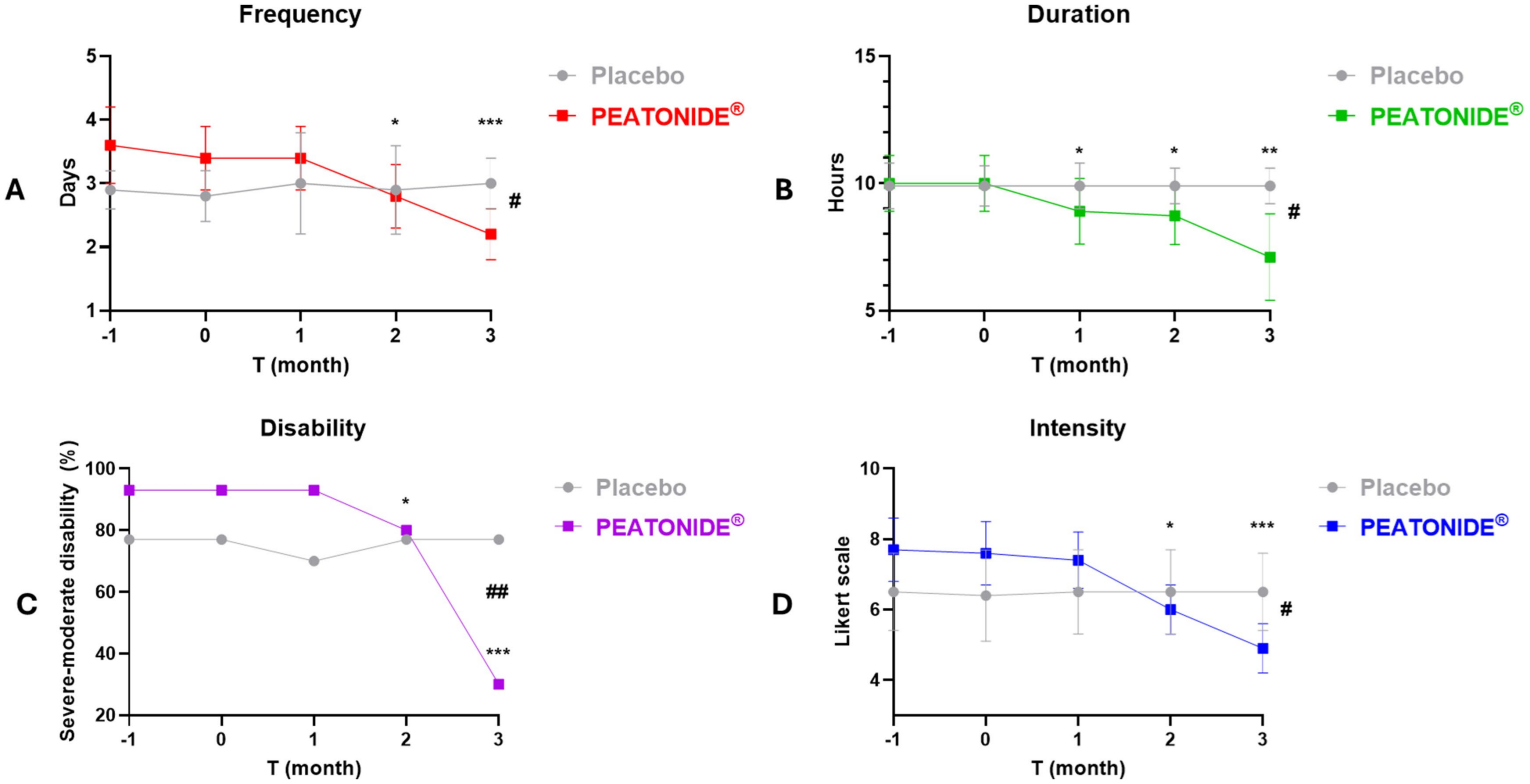
Graphical representation of migraine attack trends (e.g., duration, intensity, frequency and disability) in PEATONIDE^®^ and placebo groups in the 2 months run-in period (T-1 and baseline T0) and during the 3 months of treatment (T1 – T2 – T3). Duration (hours per day) and frequency (MMDs) of migraine attacks were summarized in averages, intensity of migraine attacks was rated on a 10-point Likert scale, moderate–severe disability was summarized as % incidence. Different symbols reveal significant differences of PEATONIDE^®^ group compared to run-in baseline period T0 (* *p* < 0.05 T3 *vs* baseline T0; **p < 0.01 T3 *vs* baseline T0; ***p < 0.001 T3 *vs* baseline T0) and compared to placebo group (^#^*p* < 0.05 T3 PEATONIDE^®^ group *vs* T3 placebo group; ^##^*p* < 0.01 T3 PEATONIDE^®^ group *vs* T3 placebo group; ^###^*p* < 0.001 T3 PEATONIDE^®^ group *vs* T3 placebo group).

Migraine preventive treatment has the primary efficacy outcome of reducing the frequency and intensity of migraine attacks, making them milder and better tolerated. Therefore, the aim of therapy is the reduction of severity and frequency by at least 50%, reducing the use of analgesic drugs and improving the quality of life of patients (41). In our study, 27% (*n* = 8) of all patients treated with PEATONIDE^®^ had a > 50% reduction in MMDs after 3 months of treatment. The study population primarily consisted of LFEM patients, with a baseline average of 3.4 ± 0.5 MMDs (95% CI 3.21–3.59). Over the 3 months treatment period, the number of MMDs decreased to 2.2 ± 0.4 MMDs (95% CI 2.05–2.35; *p* < 0.001 T3 *vs* baseline T0) (Table 1, Figure 2A). In contrast, no significant variation in MMDs was observed in the placebo group (3.0 ± 0.4 MMDs, 95% CI 2.85–3.15) after 3 months of administration. Similar to the decrease in the number of MMDs, treatment with PEATONIDE^®^ determined a reduction in analgesic medication use. In the treated group, 25% (*n* = 7) of patients had a > 50% reduction in the duration of migraine attacks after 3 months of treatment, with an overall reduction from 10.0 ± 1.1 h (95% CI 9.59–10.41) to 7.1 ± 1.7 h (95% CI 6.47–7.73; *p* < 0.01 T3 *vs* baseline T0) (Table 1, Figure 2B). In contrast, no significant variations of these parameters were observed in the placebo group, with a migraine duration of 9.9 ± 0.7 h (95% CI 9.64–10.16). In addition, PEATONIDE^®^ (7.1 ± 1.7 h; 95% CI 6.47–7.73) allowed a significant reduction of migraine attack duration compared to placebo group (9.9 ± 0.7 h; 95% CI 9.60–10.20) after 3 months of treatment (*p* < 0.05 T3 PEATONIDE^®^ group *vs* T3 placebo group) (Table 1, Figure 2B). Regarding migraine-related disability, the proportion of patients treated with PEATONIDE^®^ experiencing moderate to severe disability decreased from 93% (95% CI 91.13–94.87) to 30% (95% CI 26.27–33.73; *p* < 0.001 T3 *vs* baseline T0) (Table 1, Figure 2C). In comparison, the results obtained during the three-month monitoring in the placebo group and the baseline results of the treated group showed an incidence >75% of moderate–severe disability (Figure 2). This measurement aligns with the incidence of severe–moderate disability reported in the literature for the majority of migraine patients, as reported by the Migraine Atlas report. Furthermore, PEATONIDE^®^ allowed a significant reduction of severe–moderate disability incidence compared to placebo group after three months of treatment (30 and 77%, respectively; *p* < 0.01 T3 PEATONIDE^®^ group *vs* T3 placebo group) (Table 1, Figure 2C). The pain intensity scale score was reduced from 7.6 ± 0.9 points (95% CI 7.26–7.94) to 4.9 ± 0.7 points (95% CI 4.64–5.16) with a significant reduction of 2.7 points in PEATONIDE^®^ group (95% CI 2.3–3.2; *p* < 0.001 T3 *vs* baseline T0) after 3 months of treatment (Table 1, Figure 2D). In the placebo group, the pain intensity score was assessed to 6.5 ± 1.1 points (95% CI 6.09–6.91), without significant variation compared to baseline. Moreover, PEATONIDE^®^ (4.9 ± 0.7 point; 95% CI 4.64–5.16) allowed a significant reduction in migraine intensity compared to placebo (6.5 ± 1.1 point; 95% CI 6.09–6.91) group after three months of treatment (*p* < 0.05 T3 PEATONIDE^®^ group *vs* T3 placebo group) (Table 1, Figure 2D).

### 3.2 Efficacy of PEATONIDE^®^ on associated symptoms of migraine attacks

**Figure 3:**
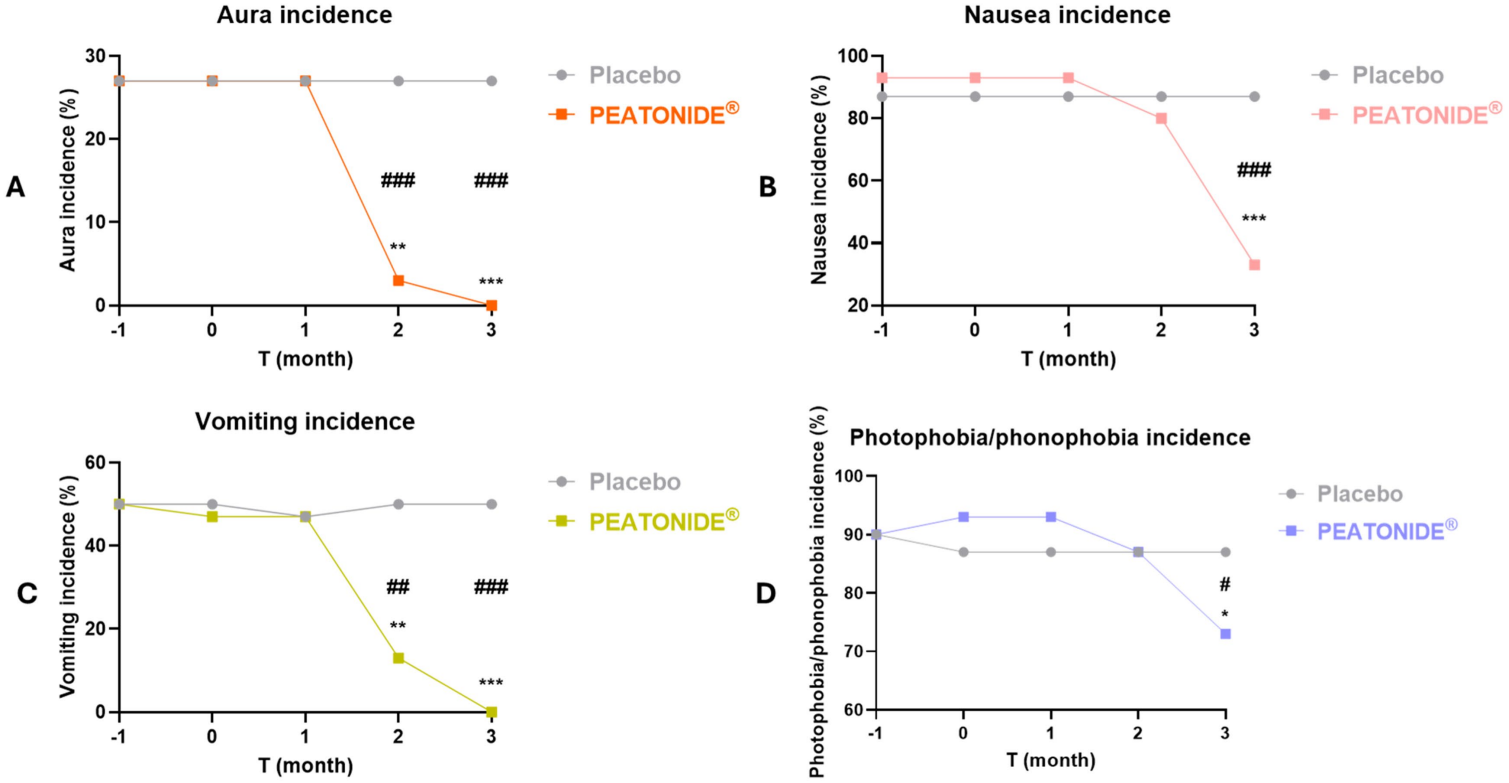
Aura, nausea, vomiting and photophobia/phonophobia incidences (%) of placebo and PEATONIDE^®^ groups in the 2 months run-in period (T-1 and baseline T0) and during the 3 months of treatment (T1 – T2 – T3). Different symbols reveal significant differences of PEATONIDE^®^ group compared to run-in baseline period T0 (*p < 0.05 T3 *vs* baseline T0; **p < 0.01 T3 *vs* baseline T0; ***p < 0.001 T3 *vs* baseline T0) and compared to placebo group (^#^*p* < 0.05 T3 PEATONIDE^®^ group *vs* T3 placebo group; ^##^*p* < 0.01 T3 PEATONIDE^®^ group *vs* T3 placebo group; ^###^*p* < 0.001 T3 PEATONIDE^®^ group *vs* T3 placebo group).

All patients treated with PEATONIDE^®^ did not experience migraine aura and reported a significant improvement in migraine-associated symptoms (e.g., photophobia, phonophobia, nausea, vomiting). All patients have observed a remission of aura events in the treated group after 3 months of treatment (27, 95% CI 25.88–28.12; *p* < 0.001 T3 *vs* baseline T0) (Table 1, Figure 3A). Aura incidence was 27% (95% CI 25.88–28.12) at baseline in both PEATONIDE^®^ and placebo groups. A significant reduction of aura incidence was observed in PEATONIDE^®^ group compared to placebo group after 2 months (1, 95% CI 0.63–1.37; *p* < 0.001 T2 PEATONIDE^®^ group *vs* T2 placebo group) and 3 months of treatment (27, 95% CI 25.88–28.12; *p* < 0.001 T3 PEATONIDE® group *vs* T3 placebo group) (Table 1, Figure 3A). The aura incidence is associated with greater severity of the study population (5, 42), consistent with the high severe–moderate disability observed in the study with an incidence >75% (Table 1). Vomiting, nausea, and photophobia/phonophobia incidences were reduced, respectively, by 100% (95% CI 99–100; *p* < 0.001 T3 *vs* baseline T0), 60% (95% CI 49–81; *p* < 0.001 T3 *vs* baseline T0), and 20% (95% CI 15–32; *p* < 0.05 T3 *vs* baseline T0) in PEATONIDE^®^ group after 3 months of treatment (Table 1, Figures 3B–D). In addition, PEATONIDE^®^ allowed a significant reduction of vomiting and nausea (*p* < 0.001 T3 PEATONIDE^®^ group *vs* T3 placebo group) and photophobia/phonophobia (*p* < 0.05 T3 PEATONIDE^®^ group *vs* T3 placebo group) incidences compared to placebo group after 3 months of treatment (Table 1, Figures 3B–D).

## 4 Discussion

The present study provides evidence supporting the efficacy of PEATONIDE®, a combination of PEA and melatonin, in reducing migraine frequency, intensity, and associated disability symptoms. Our findings demonstrate that a three-month supplementation with PEATONIDE® significantly improved key migraine parameters in patients with low-frequency episodic migraine (LFEM). The formulation was well tolerated, with no reported adverse effects, highlighting its potential as a safe preventive strategy for migraine management. PEATONIDE® formulation was based on an innovative hydrodispersible combination of PEA (1,200 mg) and melatonin (0.2 mg) in a ratio 6,000:1. The selected PEA higher dosage compared with melatonin also results from its lower solubility and bioaccessibility in gastrointestinal fluids (PEA: 1.6%; melatonin: 36%) (20). A key outcome was the significant reduction in MMDs after 3 months of PEATONIDE^®^ treatment (2.2 ± 0.4 MMDs, 95% CI 2.05–2.35) compared to placebo group (3.0 ± 0.4 MMDs, 95% CI 3.21–3.59; *p* < 0.05 T3 PEATONIDE^®^ group *vs* T3 placebo group) (Table 1, Figure 2A). Similar health benefits were observed in migraine attack duration, which decreased from 10.0 ± 1.1 h (95% CI 9.59–10.41) to 7.1 ± 1.7 h (95% CI 6.47–7.73; *p* < 0.01 T3 vs. baseline T0), and in pain intensity, which was reduced by 7.6 ± 0.9 points (95% CI 7.26–7.94) to 4.9 ± 0.7 points (95% CI 4.64–5.16) on a 10-point Likert scale (*p* < 0.001 T3 vs. baseline T0). PEATONIDE^®^ treatment was well tolerated reduced severity of and migraine attacks after 3 months of treatment with less intensive and lower daily disability correlated to migraine. This result was confirmed by the significant reduction of severe–moderate disability and pain intensity score. These factors are of high interest for the development of supplements for migraine prevention, given the impact this condition has on the individual, with limitations in work and social activities, reducing quality of life (7). An important finding is that none of the patients in the PEATONIDE^®^ group experienced adverse effects attributable to the treatment. These results are consistent with previous studies on PEA, which have shown it to be well-tolerated by patients. In addition, another relevant factor is that the subjective perception of patients is favorable to treatment with the formulation. PEATONIDE^®^ significantly reduced the incidence of associated symptoms such as nausea, vomiting, and photophobia/phonophobia, with a complete remission of aura episodes in treated patients. The placebo group, as expected, did not show significant variations in primary outcomes such as MMDs or attack duration, despite some slight fluctuations. Moreover, factors such as patient monitoring, adherence to daily headache diaries, and the overall study environment might have contributed to the mild fluctuations observed in the placebo group. Nonetheless, the statistically and clinically significant differences between the PEATONIDE® and placebo groups reinforce the reliability of our findings and confirm that the observed effects are attributable to the treatment rather than non-specific responses.

Despite the promising results, this study has some limitations. The LFEM patient population might not fully represent a broader range of migraine patients, including high-frequency episodic migraine (HFEM). To confirm the efficacy of PEATONIDE^®^ in a broader migraine population, future studies should include patients with a more balanced range of migraine frequencies and in HFEM patients. In addition, our findings suggest a possible reduction in aura incidence following PEATONIDE^®^ treatment. However, given the small sample size of patients experiencing aura, these results should be interpreted with caution and warrant further investigation in larger cohorts to confirm the efficacy in broader migraine population. Another potential limitation of the study is the reliance on self-reported data about lifestyle habits and dietary intake. Although patients were instructed to maintain their usual routines and to avoid other supplements or medications that might affect migraine frequency, individual variations in diet, physical activity, and sleep patterns could represent potential confounding factors. While baseline assessments were conducted to account for these factors, the absence of objective lifestyle monitoring (e.g., dietary logs, actigraphy for sleep assessment) represents a methodological constraint. Future studies should integrate more precise tracking methods to better control for potential confounding variables. All patients in the two groups have taken analgesic drugs during MMDs. Patients were allowed to take moderately effective drugs such as acetaminophen or NSAIDs but not more potent drugs like triptans. This restriction was intended to better evaluate the impact of PEATONIDE^®^ during the acute phase of migraines. Although our study included both male and female patients, it did not specifically analyze sex-related differences in response to PEATONIDE^®^. Given the known influence of hormonal fluctuations on migraine pathophysiology, mainly for estrogen variability of women, it would be valuable to assess whether sex-based differences impact treatment efficacy (49). Future studies could incorporate stratified analyses to evaluate whether hormonal factors influence the sex differences in migraine response after PEATONIDE^®^ treatment (7).

The migraine burden is defined as the cumulative physical, emotional, and social effects that migraine take on patients, including both the acute attacks and the time between them (43). Therefore, the contextual reduction in parameters as frequency, duration, intensity, disability, and migraine-related symptoms incidence suggests a direct reduction of migraine burden and an improvement of patients quality of life. Moreover, although migraine burden was not directly evaluated by specific questionnaires (e.g., HIT-6 and MIDAS), an estimation of the positive effect of PEATONIDE^®^ treatment can be obtained from data set shown in Table 1.

The health-benefits effects of PEA and melatonin-based supplements agreed with previously published studies. Artukoglu et al. (44) conducted a meta-analysis from 10 published works collecting data from 786 cases and 512 controls. Although the limitation of the low studies homogeneity, the study concluded that PEA seemed to be effective in chronic pain treatment due to different substances (44). Similar conclusions were reached by Gabrielsson et al. (13) in a review of clinical trials related to pain treatment with PEA. In both studies, PEA displayed an excellent tolerability profile, with almost no undesirable side effects, except for isolated cases of gastrointestinal disorders (13, 44). In a previously published clinical trial, the treatment with 1,200 mg of ultramicronized PEA decreased of MMDs from 3.1 ± 0.6 days to 2.0 ± 1.0 days (*p* = 0.001). In addition, the reduction of intensity and duration of migraine attacks was significant after 2 months and was maintained after 3 months of treatment (38). These scores agreed with our clinical data, which was performed using the same PEA dosage. Moreover, Hernández et al. (7) determined that a formulation containing 200 mg of PEA displayed a 36% reduction in patients with a severe–moderate disability on the MIDAS scale, and a 50% reduction of major or severe impact according to the HIT-6 scale after 3 months of treatment. The higher 68% reduction observed in our study is likely due to the sixfold higher PEA dosage (1,200 mg) compared to the 200 mg formulation used in the referenced study, suggesting that a higher dosage provides enhanced clinical benefits.

Although migraine has a complex pathophysiology, linking structures of the central and peripheral nervous system, there is evidence to support a primary role of inflammation in migraine genesis (31). Several studies have identified PEA’s central role in regulating the effects of PPAR receptors in migraine disorders (15). In addition, PEA has demonstrated to act directly by inhibiting mast cells and reducing their production of pro-inflammatory substances. PEA acts on microglia by regulating the transmission of the pain impulse upstream to the CNS, where it also has a positive effect on CB1 and CB2 receptors in neurons, therefore modifying the pain processing response (15, 16). On the other hand, melatonin regulates other pathways involved in migraine attacks. Recent studies highlighted the key role played by the neurotransmitter PACAP in the regulation of migraine in the hypothalamic region (34, 45). PACAP induces vasodilation via a cAMP-dependent mechanism and regulates mast cell degranulation. In addition, PACAP is secreted during mast cell degranulation, controlling neurotransmitters release by an autocrine mechanism (46). Melatonin inhibits the activity of the neurotransmitter PACAP in the hypothalamus, reducing the vasodilation involved in migraine attacks (33, 47).

A common mechanism of action of PEA and melatonin in migraine attack control is related to the regulation of mast cell degranulation, which controls the migraine neuroinflammatory response. Therefore, the combination of the two molecules could increase the potential anti-migraine activity through a synergistic effect. Mast cells degranulation is regulated by several neuroinflammatory mediators, including CGRP, which is released by neurons of the trigeminal nerve and the trigeminal ganglion and represents the main neurotransmitter involved in migraine vasodilation (34). The combination of PEA and melatonin inhibits mast cell degranulation, reducing vasodilation and thus the symptoms associated with migraine (19, 20, 37). Supplementation with PEATONIDE^®^ showed a lag time of about 1 month to observe the antimigraine activity (Figures 2, 3). The one-month onset period could be related to the mechanism of mast cells degranulation inhibition. Moreover, mast cells degranulation inhibitors (e.g., cromoglycate sodium, nedocromil) typically require several days or weeks to produce an appreciable pharmacological activity (48).

## 5 Conclusion

The identification of safe and efficient treatment represents a crucial research focus for the development of new formulations for episodic migraine management. Therefore, the combination of PEA and melatonin, two molecules with a well-known antimigraine activity, represents a valuable tool to counteract migraine-related symptomatology. In our study, the efficacy of the nutraceutical formulation PEATONIDE^®^ in the management of episodic migraine was observed by a randomized, three-month, double-blind, placebo-controlled trial. The formulation was effective both in the reduction of frequency, intensity, duration, and grade of disability of migraine attacks and in mitigation of migraine-related symptomatology (e.g., aura, nausea, vomiting, photophobia/phonophobia). In addition, the higher prevalence of aura in the study population makes these results particularly relevant for patients with a similar clinical profile. Overall, this study supports the use of the supplement PEATONIDE^®^ as an alternative therapy for migraine attacks treatment and confirms the future role of PEA and melatonin-based nutraceuticals in migraine management. Despite the promising results, additional follow-up studies with larger sample sizes are needed to further validate the observed efficacy and safety of PEATONIDE^®^. Expanding the study population to include individuals with HFEM and chronic migraine could provide a more comprehensive understanding of its applicability. Moreover, larger-scale, multicenter trials with longer follow-up periods and objective monitoring methods will be essential to confirm the robustness of these results and optimize the clinical application of PEATONIDE^®^ in migraine prevention.

## Data Availability

The raw data supporting the conclusions of this article will be made available by the authors, without undue reservation.

## Footnotes

^1^ www.isrctn.com

^2^ https://www.isrctn.com/ISRCTN52370199
